# Intravital Microscopy Reveals Endothelial Transcytosis Contributing to Significant Tumor Accumulation of Albumin Nanoparticles

**DOI:** 10.3390/pharmaceutics15020519

**Published:** 2023-02-03

**Authors:** Guoguang Wei, Sihang Zhang, Sheng Yu, Wei Lu

**Affiliations:** Key Laboratory of Smart Drug Delivery, Ministry of Education & State Key Laboratory of Molecular Engineering of Polymers, School of Pharmacy & Minhang Hospital, Fudan University, 826 Zhangheng Road, Shanghai 201203, China

**Keywords:** the enhanced permeability and retention (EPR) effect, albumin-bound nanoparticles, endothelial transcytosis, near-infrared-II (NIR-II), intravital microscopy

## Abstract

The principle of enhanced permeability and retention (EPR) effect has been used to design anti-cancer nanomedicines over decades. However, it is being challenged due to the poor clinical outcome of nanoparticles and controversial physiological foundation. Herein, we use a near-infrared-II (1000–1700 nm, NIR-II) fluorescence probe BPBBT to investigate the pathway for the entry of human serum albumin-bound nanoparticles (BPBBT-HSA NPs) into tumor compared with BPBBT micelles with phospholipid-poly (ethylene glycol) of the similar particle size about 110 nm. The plasma elimination half-life of BPBBT micelles was 2.8-fold of that of BPBBT-HSA NPs. However, the area under the BPBBT concentration in tumor-time curve to 48 h post-injection (AUC_tumor0→48h_) of BPBBT-HSA NPs was 7.2-fold of that of BPBBT micelles. The intravital NIR-II fluorescence microscopy revealed that BPBBT-HSA NPs but not BPBBT micelles were transported from the tumor vasculature into tumor parenchyma with high efficiency, and endocytosed by the tumor cells within 3 h post-injection in vivo. This effect was blocked by cross-linking BPBBT-HSA NPs to denature HSA, resulting in the AUC_tumor0→48h_ decreased to 22% of that of BPBBT-HSA NPs. Our results demonstrated that the active process of endothelial transcytosis is the dominant pathway for albumin-bound nanoparticles’ entry into tumor.

## 1. Introduction

In the past several decades, nanotechnology has achieved great progress and success in biomedical sciences, especially in the area of cancer research. To date, numerous research articles have reported substantial effects on tumor regression with a wide variety of nanomedicine [1,2,3,4,5]. A great number of anticancer nanomedicine candidates have been submitted for preclinical investigation [6,7,8]. Nevertheless, only a few have been approved by regulatory agencies [9]. The biggest obstacle to clinical translation is the low tumor delivery efficiency (<0.7%) of the nanomedicine [10].

The EPR effect is the central rationale for the tumor-targeted delivery of the nanoparticles [11,12]. The fundamental features of the EPR effect rely on the premise that nanoparticles are able to pass through the gaps between tumor vascular endothelial cells into the tumor region. Therefore, the hyperpermeable tumor vasculature contributes to the increased extravasation of nanoparticles, while the absence of lymphatic vessels in tumor elongates the retention time of these nanocarriers. Based on this theory, appropriate size range and long circulation time have long been the golden principles to design nanodrug delivery systems for cancer therapy [13,14,15].

However, with the increasing cases of the failure of nanodrugs to present the improved anticancer efficacy in clinical trials, the principle of EPR effect is suffering from challenges and controversy [16,17,18]. A recent study by Chan’s group showed that the inter-endothelial gaps occur rarely and are not the major factor responsible for the nanoparticle transportation into tumors [17,19]. By using gold nanoparticles, they shed light on that 97% of the nanoparticles utilize active process to enter tumors through endothelial cells [17]. So, what is the key factor influencing nanoparticles’ entry into tumors: active transport via endothelial transcytosis or passive process through the EPR effect? It is imperative to figure out this question on the dosage forms of commercially available nanomedicine besides gold nanoparticles, since the material composition and other parameters may affect the biological process and tumor entrance of the nanoparticles [16,20].

Albumin nanoparticles such as nanoparticle albumin-bound paclitaxel (nab-paclitaxel) are widely used as monotherapy or combination therapy of metastatic breast cancer, non-small-cell lung cancer (NSCLC) and advanced pancreatic cancer (PC) [21]. The tumor delivery of nab-paclitaxel has been thought to be an active process, through which the nanoparticles can bind gp60 expressed on the tumor vascular endothelium facilitating caveolin-1-mediated transcytosis [22,23]. On the other hand, micellular drug delivery systems such as the methoxy poly(ethylene glycol)-*b*-poly(D,L-lactide) (mPEG-*b*-PDLLA) micelles loading docetaxel (Nanoxel^®^ M) are used for the treatment of recurrent or metastatic head and neck squamous cell carcinoma [24,25]. The micelles with long-blood circulation characteristic have been considered to enter into the tumor through the EPR effect [26]. These reports suggest that the material composition (albumin versus polymer or phospholipid) and surface chemistry (with versus without PEGylation) of the nanoparticles play a critical role in the mechanism of active transcytosis or passive extravasation into tumors [27].

Fluorescence microscopy is widely applied to biological application, especially biomedical imaging. The unique and/or tunable optical property of nanoparticles has exhibited versatility for the fluorescence bioimaging. The photoluminescent silicon nanoprobes with multiplex emissions have been reported as cell-selective agents for diagnosis of HeLa cells and the cell viability under bioimaging [28]. Nanoparticles such as polymer dots, quantum dots, silicon nanorods and gold nanoparticles have been developed as dual-emission ratiometric fluorescence sensors. With the self-calibration characteristics, these nanomaterials greatly improve the signal-to-noise ratios and the accuracy of molecular detection and imaging [29]. Recently, a variety of nanomaterials and probes have been designed with the fluorescence emission spanning the second near-infrared (NIR-II) window (1000–1700 nm) [30]. In comparison with NIR-I (650–950 nm) fluorescence, NIR-II fluorescence offers deeper tissue optical imaging with increased signal-to-background ratio, owing to reduced photon scattering, absorption and tissue autofluorescence interference [31]. Due to these advantages, the one-photon confocal imaging system based on NIR-II fluorescence has been developed to realize the imaging of brain vasculatures up to ~1.3 mm of depth [32].

Herein, we used a lipophilic NIR-II fluorescence molecule, BPBBT, as a model drug to investigate the tumor entrance of the albumin-bound nanoparticles. Similar as paclitaxel, BPBBT exhibits high binding affinity to human serum albumin (HSA), allowing to formulate stable HSA-bound BPBBT nanoparticles (BPBBT-HSA NPs) [33]. To compare the active transcytosis of the albumin-bound nanoparticles with the passive transport of the PEGylated micelles through the EPR effect, the BPBBT micelles of the similar particle size are prepared with N-(Carbonyl-methoxy polyethylene glycol 2000)-1,2-distearoyl-sn-glycerol-3-phosphoethanolamine (DSPE-PEG_2000_). BPBBT-HSA NPs are additionally subjected to surface cross-linking (BPBBT-CL-HSA NPs) in order to assess the effect of surface chemistry of the albumin-bound nanoparticles on their active transport efficiency. BPBBT possesses high quantum yield of NIR-II fluorescence peaked at 1065 nm [34]. By utilizing a home-built intravital NIR-II fluorescence microscopic system, we compared the process of the in vivo tumor-targeting effect among the three different BPBBT nanoparticles (BPBBT NPs), to find out the key factor influencing nanoparticles’ entry into the tumor.

## 2. Materials and Methods

### 2.1. Materials

HSA was purchased from Instituto Grifols, S.A. (Barcelona, Spain). DSPE-mPEG_2000_ was purchased from Advanced Vehicle Technology Pharmaceutical Co., Ltd. (Shanghai, China). 4′,6-Diamidino-2-phenylindole (DAPI) was purchased from Meilun Biotechnology Co., Ltd. (Dalian, China). Rat anti-mouse CD31 antibody (PECAM-1; 102401) was purchased from Biolegend (San Diego, CA, USA). Dichloromethane, tetrahydrofuran and other analytical reagents were purchased from Sinopharm Chemical Reagent Co., Ltd. (Shanghai, China). 1,1′-Dioctadecyl-3,3,3′,3′-tetramethylindodicarbocyanine,4-chlorobenzenesulfonate salt (DiD) cell-labeling solution was purchased from ThermoFisher Scientific (Waltham, MA, USA).

### 2.2. Cells and Animals

Mouse colon cancer CT-26 cell line stably expressing luciferase CT26-Luc (CT26.WT-Fluc-Neo) was obtained from Imanis Life Sciences (Rochester, MN, USA). Cells were cultured in RPMI 1640 supplemented with 10% (*v*/*v*) fetal bovine serum (FBS, HyClone, Logan, UT, USA), penicillin (100 µg/mL) and streptomycin (100 µg/mL) at 37 °C and 5% CO_2_.

BALB/c mice (male, 6–8 weeks) were ordered from Shanghai Lingchang Biological Co., Ltd. (Shanghai, China) and housed under the specific pathogen-free (SPF) conditions with free access to food and water. All animals were allowed to adapt to the environment for at least one week before experiments. All animal experiments were performed under the guidance of Institutional Animal Care and Use Committee (IACUC) of School of Pharmacy, Fudan University.

### 2.3. Preparation of BPBBT Micelles

BPBBT micelles were prepared by adding 0.5 mL of tetrahydrofuran (THF) solution containing 0.5 mg of BPBBT and 1 mg of DSPE-PEG_2000_ to 5 mL of distilled water. The mixture was sonicated with ultrasonic cell crusher (Scientz, Ningbo, China) for 2 min under ice bath, followed by stirring for 4 h at 40 °C. The solution was filtered (0.22 μm, Millipore, Burlington, MA, USA) and washed three times with an ultrafiltration tube (MWCO 100 kDa, Merck, Rahway, NJ, USA).

### 2.4. Preparation of BPBBT-HSA NPs or BPBBT-CL-HSA NPs

BPBBT (1 mg/mL) was dissolved in dichloromethane. HSA (2 mg/mL) was diluted with phosphate-buffered saline (PBS). The HSA solution was mixed with BPBBT solution and the mixture was homogenized for 2 min with a homogenizer (Jinxin, Shanghai, China) to form a crude emulsion. The emulsion was transferred and sonicated with ultrasonic cell crusher (Scientz, China) for 5 min under ice bath. The organic solvent was rapidly removed at reduced pressure for 10 min. The BPBBT-HSA NPs were obtained by filtration (0.22 μm, Millipore, USA) and washed three times using an ultrafiltration tube (MWCO 100 kDa, Merck, USA). For the preparation of BPBBT-CL-HSA NPs, the prepared BPBBT-HSA NPs were cross-linked by the addition of 8% glutaraldehyde solution (1.175 μL/mg HSA) followed by stirring for 24 h at room temperature [35,36,37]. The BPBBT-CL-HSA NPs solution was washed three times using an ultrafiltration tube (MWCO 100 kDa, Merck, USA).

### 2.5. Characterization of Different BPBBT NPs

The particle sizes and zeta potential of the three types of BPBBT NPs, i.e., BPBBT-HSA NPs, BPBBT-CL-HSA NPs and BPBBT micelles, were measured by a dynamic light scattering (DLS) instrument (Malvern Nanozetasizer, Worcester, UK). Morphology of these BPBBT NPs was observed by transmission electron microscope (TEM) (FEI Tecnai G2 20 TWIN, Hillsboro, OR, USA). To improve the contrast, BPBBT-HSA NPs and BPBBT-CL-HSA NPs were visualized with negative staining (2% phosphotungstic acid). For the stability test, BPBBT NPs were added to 10% FBS (*w*/*v*) at 37 °C to give a final concentration of BPBBT (0.6 mg/mL). DLS analysis was performed at different time points following the mixture. Ultraviolet-visible-near-infrared (UV-vis-NIR) absorption spectra were recorded on a Lambda 365 spectrophotometer (PerkinElmer, Waltham, MA, USA). The NIR-II fluorescence emission spectra were measured using a fluorescence spectrometer (PTI QM40, Holland, OH, USA).

The UV-vis-NIR absorbance at 808 nm was used to quantify the concentration of BPBBT in the nanoparticles. The drug loading efficiency (DL) and encapsulation efficiency (EE) of BPBBT in the nanoparticles were calculated using the following equation.

EE% = Weight of the encapsulated BPBBT/Weight of BPBBT added × 100%

DL% = Weight of the encapsulated BPBBT/Weight of nanoparticles × 100%

### 2.6. In Vitro Cytotoxicity of Different BPBBT NPs

NIH 3T3 cells obtained from the American Type Culture Collection (ATCC, Manassas, VA, USA) were used for the in vitro cytotoxicity evaluation of different BPBBT NPs via methyl thiazolyl tetrazolium (MTT) assay. The cells (1 × 10^4^ per well) were cultured in 96-well microplates for 24 h before the experiment. The cells were incubated with a series of concentrations of BPBBT NPs for 24 h. Replaced with fresh culture medium, 20 μL of MTT solution (5 mg/mL) was added to each well. After 2 h of incubation, the supernatant was replaced with 150 μL of dimethyl sulfoxide (DMSO). The absorbance was measured at 570 nm via Bio-Rad 550 microplate reader (Bio-Rad, Hercules, CA, USA).

### 2.7. Biodistribution Analysis and Pharmacokinetics Study

BALB/c mice bearing CT26-Luc orthotopic tumor were established according to our previous reported method [33]. After tumor inoculation for 7 d, the mice were intravenously (i.v.) injected with different BPBBT NPs (20 mg/kg of BPBBT). The mice were euthanized at 1, 12, 24 or 48 h post-injection (*n* = 3). Tumor foci or major organs were collected and weighed. Then, PBS (three times of the tissue weight) was added and homogenized by a tissue lyser (Jinxin, China) for 3 min. The procedure of the extraction of BPBBT and quantitative analysis of its concentration by measuring the NIR-II fluorescence intensity at 1065 nm following our previous method [33]. The blank tissue samples were added with predetermined concentration of different BPBBT NPs, and received the same treatment for the calibration and validation test. The area under the BPBBT concentration in tumor-time curve to 48 h post-injection (AUC_tumor0→48h_) was calculated by the trapezoidal method based on the measured BPBBT concentration in tumor at different time points post-injection.

For the pharmacokinetics study, the tumor-bearing mice were i.v. injected with different BPBBT NPs (20 mg/kg of BPBBT) and euthanized at 1, 12, 24 or 48 h post-injection (*n* = 3). The blood was collected at different time points (0.5, 1, 2, 4, 8, 12, 24, 48 or 72 h). After centrifugation at 4000 rpm for 10 min, the plasma was collected for further analysis. The plasma samples (10 μL) of 0.5, 1, 2, 4, 8 and 12 h were collected from the same mice and diluted into 100 μL with PBS. The plasma samples (100 μL) of 24, 48 or 72 h were collected from additional three mice per group for each time point. The pharmacokinetic parameters were calculated using Via PKSolver software (China Pharmaceutical University). The plasma concentration of BPBBT of the three nanoparticles fit the two-compartmental model since the R-square values of all the groups were above 0.99. Therefore, the pharmacokinetic parameters including distribution half-life (*t*_1/2α_), elimination half-life (*t*_1/2β_), clearance (*Cl*), area under the concentration-time curve to the least measurable concentration (AUC_0→t_) and steady-state volume of distribution (*V*_ss_) were calculated using the two-compartmental model.

### 2.8. Intravital Microscopic Imaging

A home-built intravital NIR-II/Red dual-channel fluorescence microscopy was set up according to our previous reported method [38]. The NIR-II fluorescence of BPBBT passed through a 1000 nm long pass filter was collected with an InGaAs camera (SW640-T, TEKWIN, Xi’an, China). The emission light of DiD passed through a 670–780 nm band pass filter was collected with a scientific complementary metal-oxide semiconductor camera (PCO edge 4.2, PCO AG, Kelheim, Germany).

The abdominal skin hair of mice bearing CT26-Luc orthotopic tumor was removed and the abdominal skin was sanitized using 0.5% (*w*/*v*) iodophor. Under the anesthesia, a small midline incision (3–5 mm) was cut in the skin of lower abdomen to expose the tumor foci on the cecum. The tumor foci were then immobilized. Additionally, the water immersion lens was adjusted for the appropriate distance above the observation window. The exposed abdominal tissues were covered with warm PBS-soaked gauze to prevent dehydration. The mice were i.v. injected with different BPBBT NPs (20 mg/kg of BPBBT) immediately before the imaging. For the investigation of tumor cell targeting effect of the nanoparticles, the mice were inoculated with the DiD-stained CT26-Luc cells for 7 d. The imaging started at 1 h post-injection of the BPBBT NPs.

The images were processed by Photoshop CC 2018 (Adobe) for pseudo-coloring on different fluorescent channels. The movies were compiled by Premiere Pro 2020 (Adobe) with the processed images. The diffusive capacity of the nanoparticles was evaluated by quantifying the fluorescence intensities at a distance of 50 μm from blood vessel to tumor parenchyma. The fluorescence intensity at the indicated time point was normalized to that of the blood vessel at 0 h after injection. At each time point, a curve of the normalized fluorescence intensity over diffusive distance was plotted. Using GraphPad Prism 9.0 software, the area under the normalized fluorescence intensity-distance curve (AUNFIC) was calculated by integration.

### 2.9. Immunofluorescent Staining

The CT26-Luc colon cancer tumor-bearing mice were i.v. injected with different BPBBT NPs (20 mg/kg of BPBBT). After 2 h, the mice were euthanized. The tumor was collected and embedded in Tissue-Tek OCT compound (Sakura Finetek, Tokyo, Japan) on dry ice for cryosectioning. The tissue sections (8-μm thickness) were fixed in 4% paraformaldehyde for 10 min and blocked with 10% FBS for 30 min. Vascular endothelial cells were detected by immunofluorescence staining using the anti-CD31 antibody (1:100) followed by goat anti-rabbit Alexa Fluor 647 secondary antibody (1:200; Invitrogen, Waltham, MA, USA). The cell nuclei were counterstained with DAPI. The fluorescence micro-images of BPBBT and Alexa Fluor 647-stained vascular endothelial cells were captured by the home-built dual-channel microscopic imaging system, respectively. The fluorescence micro-images of Alexa Fluor 647-stained vascular endothelial cells and DAPI of the same area of the section was additionally captured by a spinning disk confocal super resolution microscope (SpinSR 10, Olympus, Tokyo, Japan), respectively. The fluorescence images of BPBBT, Alexa Fluor 647-stained vascular endothelial cells and DAPI-stained nuclei were merged using Adobe Photoshop following the alignment based on the image of Alexa Fluor 647-stained vascular endothelial cells. The area of vascular endothelial cells was determined by measuring the CD31-positive area via ImageJ. The endocytosed nanoparticle clusters were defined as the fluorescence of clusters completely or partially co-localized with that of vascular endothelial cells (the CD31-positive area). The extravasated nanoparticle clusters were defined as the fluorescence of clusters from 5 to 100 μm outside of each vessel according to the literature [19]. Semi-quantification of nanoparticle clusters on vascular endothelial cells were performed according to the previous report with slight modification [19].

### 2.10. Statistical Analysis

Statistical analysis was performed using Graphpad Prism 9.0. All results were presented as mean ± SD. The results were analyzed by a two-tailed Student’s *t*-test between two groups. One-way analysis of variance (ANOVA) with Dunnett’s post hoc test or two-way ANOVA with Tukey post hoc test was used for multiple groups.

## 3. Results

### 3.1. Characterization of Different BPBBT NPs

**Figure 1:**
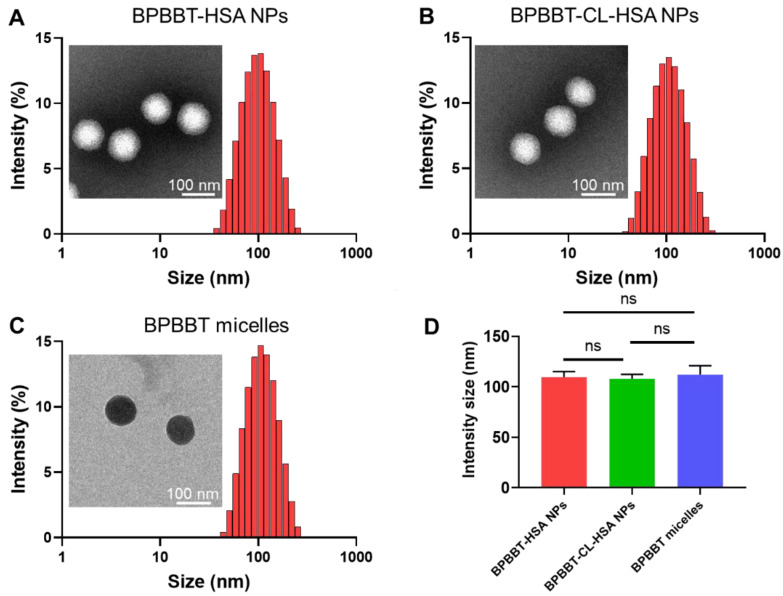
Characterization of different types of BPBBT nanoparticles. (**A**–**C**) Size distribution and TEM of BPBBT-HSA NPs (**A**), BPBBT-CL-HSA NPs (**B**) and BPBBT micelles (**C**), respectively. The samples of BPBBT-HSA NPs and BPBBT-CL-HSA NPs were negatively stained with 2% phosphotungstic acid before the TEM. (**D**) The average particle sizes of different types of BPBBT nanoparticles. Data are presented as mean ± SD (*n* = 3). ns, no significance by one-way ANOVA with a Dunnett’s post hoc test.

The hydrophobic NIR-II probe BPBBT (4, 8-Bis [4-(*N*, *N*-Bis (4-octyloxyphenyl) amino) phenyl] benzo [1,2-*c*:4,5-*c*′] bis ([1,2,5] thiadiazole) based on an electron donor-acceptor-donor (D-A-D) structure has the maximum absorption at 750 nm and possesses high quantum yield of NIR-II fluorescence peaked at 1065 nm (Figures S1 and S2). The BPBBT micelles were prepared by using the solvent evaporation method. In order to obtain the similar loading efficacy and particle size as BPBBT micelles, BPBBT-HSA NPs were prepared using a modified “nab-technology” [33]. The feeding ratio of BPBBT to HSA was increased from 1:15 to 1:2. The mixture of the HSA solution with BPBBT solution was through ultra-sonification instead of homogenization. BPBBT-HSA NPs and BPBBT micelles had similar particle size of 109.67 ± 5.41 nm and 112.03 ± 8.88 nm in diameter, and similar zeta potential of −16.97 ± 0.75 mV and −17.47 ± 0.65 mV, respectively (Figure 1, Table 1). The polymer dispersity index (PDI) of BPBBT-HSA NPs and BPBBT micelles was 0.15 ± 0.01 and 0.13 ± 0.01, respectively. The cross-linking process did not influence the average particle size or size distribution of BPBBT-HSA NPs. The particle size and PDI of BPBBT-CL-HSA NPs were 108.07 ± 4.29 nm and 0.20 ± 0.01, respectively. However, the cross-linking process reduced the zeta potential of BPBBT-CL-HSA NPs to −25.40 ± 1.91 mV, which was 8.43 mV lower than that of BPBBT-HSA NPs. The TEM confirmed the uniform and similar size distribution of all the three nanoparticles. By optimizing the formulation procedure, these nanoparticles have the similar EE% and DL% of BPBBT (Table 1). The UV-vis-NIR absorption spectra showed a 45 nm blue-shifted absorbance peak of the BPBBT NPs compared with BPBBT in THF (Figure S2A). On the other hand, the fluorescence intensity of BPBBT were significantly increased but with a slightly blue-shift of the emission peaks after the nanoparticle preparation in comparison with that of the soluble BPBBT in THF (Figure S2B). Due to the aggregation-induced emission characteristic of BPBBT [33], the enhanced fluorescence intensity in the NIR-II region of the BPBBT nanoparticles ensured the imaging sensitivity under the intravital NIR-II fluorescence microscopy. These results collectively demonstrated that BPBBT-HSA NPs, BPBBT-CL-HSA NPs and BPBBT micelles were successfully prepared with similar nanoparticle parameters including particle size, size distribution, morphology, drug loading and optical properties. Moreover, all the three types of BPBBT NPs exhibited negligible cytotoxicity to mouse embryonic fibroblasts NIH 3T3 cells following 24 h incubation at the BPBBT concentration up to 200 μg/mL (Figure S3), indicating that these BPBBT NPs were biocompatible for the in vivo bioimaging.

### 3.2. Stability of BPBBT NPs

**Figure 2:**
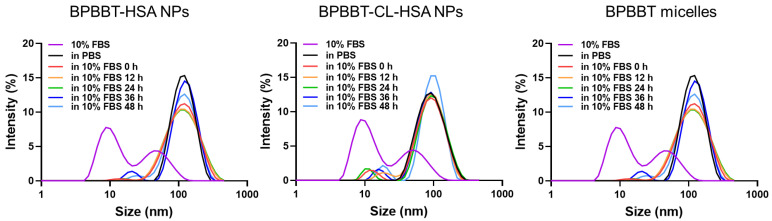
Size distribution of different types of BPBBT nanoparticles in PBS or in 10% FBS for different incubation times. The size distribution of 10% FBS was used as control.

The stability of three types of BPBBT NPs was investigated in PBS and PBS containing 10% FBS (*v*/*v*), respectively. As shown in Figure 2, the average size of all the three BPBBT NPs remained unchanged for 48 h, indicating that all BPBBT NPs were stable.

### 3.3. Pharmacokinetics and Biodistribution of Different BPBBT NPs

**Figure 3:**
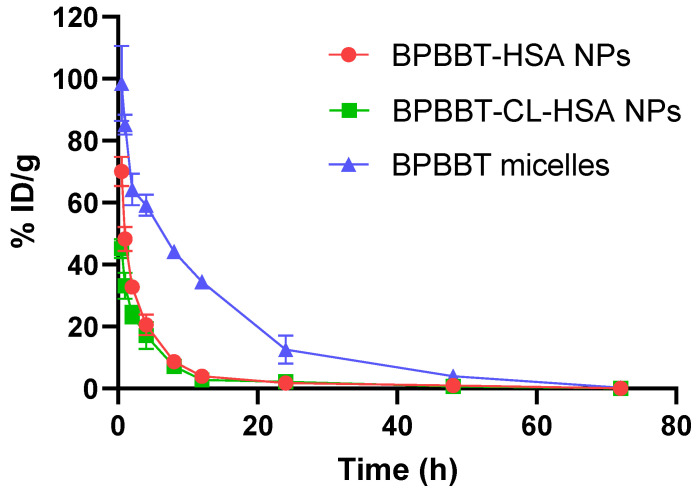
The plasma concentration-time curves of BPBBT in BALB/c mice bearing CT26-Luc orthotopic tumor after i.v. administrated with BPBBT-HSA NPs, BPBBT-CL-HSA NPs or BPBBT micelles (20 mg/kg of BPBBT). Data are presented as mean ± SD (*n* = 3).

The pharmacokinetic profiles of the three types of nanoparticles fit the two-compartment model by a linear least squares method via PKSolver. Comparative results illustrated that BPBBT-HSA NPs and BPBBT-CL-HSA NPs had similar profiles of the blood concentration-time curves in mice, indicating that cross-linking did not alter the pharmacokinetics of the HSA-bound nanoparticles (Figure 3, red and green curves). This was confirmed by the pharmacokinetic parameters without significant difference between the two groups except the steady-state volume of distribution (*V*_ss_) and distribution half-life (*t*_1/2α_) (Table 2). By contrast, the BPBBT micelles exhibited much longer blood circulation property, resulting in the plasma elimination half-life (*t*_1/2β_) 2.8-fold of that of BPBBT-HSA NPs or BPBBT-CL-HSA NPs. This result was in line with the previous report, confirming that the surface modification of micelles with poly(ethylene glycol) (PEG) elongated their blood circulation [39,40]. Consequently, the BPBBT micelles increased plasma area under concentration−time curve (AUC) by 4.3-fold and 5.7-fold in comparison with BPBBT-HSA NPs and BPBBT-CL-HSA NPs, respectively.

**Figure 4:**
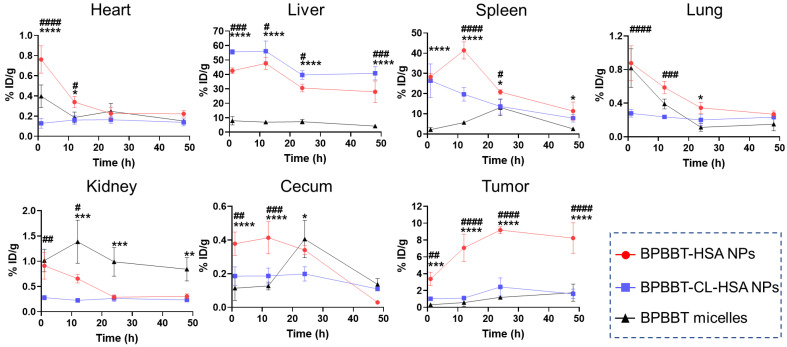
Biodistribution of BPBBT in mice bearing CT26-Luc orthotopic tumor at 1, 12, 24 or 48 h after i.v. injection of different types of BPBBT NPs (20 mg/kg of BPBBT). The amount of BPBBT was determined by measurement of its fluorescence intensity at 1065 nm extracted from the tissue. % ID/g, Percentage of the injected dose per g tissue. Data are presented as mean ± SD (*n* = 3). * *p* < 0.05, ** *p* < 0.01, *** *p* < 0.001, **** *p* < 0.0001 between the BPBBT-HSA NPs group and the BPBBT micelles group; ^#^ *p* < 0.05, ^##^ *p* < 0.01, ^###^ *p* < 0.001, ^####^ *p* < 0.0001 between the BPBBT-HSA NPs group and the BPBBT-CL-HSA NPs group, calculated via two-way ANOVA with Tukey post hoc test.

The longer blood circulation led to a significantly lower distribution of BPBBT micelles in liver and spleen than the HSA-bound nanoparticles (Figure 4), attributed to the reduced nonspecific uptake by the reticuloendothelial system (RES). Owing to the EPR effect, the longer blood circulation of BPBBT micelles resulted in a gradual increase of BPBBT in the tumor over time. However, the BPBBT concentration in tumor of BPBBT micelles was much lower than that of BPBBT-HSA NPs at 1 h (0.31 versus 3.38% ID/g), 12 h (0.57 versus 7.08% ID/g), 24 h (1.20 versus 9.19% ID/g) and 48 h (1.74 versus 8.23% ID/g) post-injection, respectively. The AUC_tumor0→48h_ of BPBBT-HSA NPs was 7.2-fold of that of BPBBT micelles, indicating that the EPR effect of the micelles counted less for the tumor-targeting efficiency. By contrast, the active process by the endothelial transcytosis contributed to significant tumor accumulation of BPBBT-HSA NPs. This was evidenced by the fact that cross-linking of the HSA carrier denatured the protein and prohibited its binding to the endothelial cells, leading to the AUC_tumor0→48h_ decreased to only 22% of that of BPBBT-HSA NPs.

### 3.4. Extravasation of BPBBT NPs by Immunofluorescence Imaging

**Figure 5:**
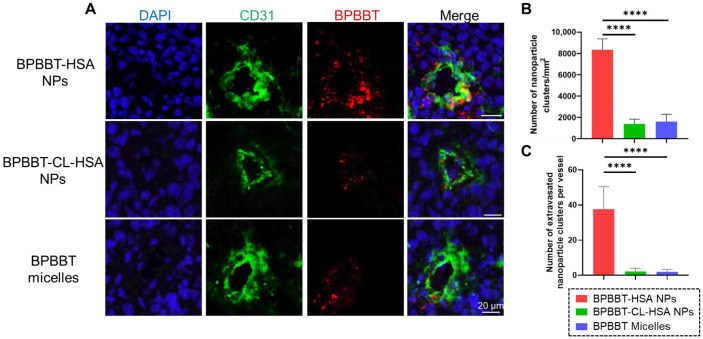
Distribution of BPBBT NPs in the tumor of mice following i.v. administration. (**A**) Representative immunofluorescence micrographs of tumor sections after i.v. injection with different types of BPBBT NPs (20 mg/kg of BPBBT) for 2 h. The endothelial cells of blood vessels were stained with anti-CD31 antibody. (**B**) Quantitative analysis of the endocytosed nanoparticle clusters per vessel area (mm^2^). (**C**) Quantitative analysis of the nanoparticle clusters in the adjacent region from 5 to 100 μm around each vessel. (**B**, **C**) Data are presented as mean ± SD, 3 tumors per group, 3 fields of views (FOVs) per tumor sample. **** *p* < 0.0001 was calculated via one-way ANOVA with a Dunnett’s post hoc test.

We next evaluated the nanoparticles transportation into tumor via immunofluorescence staining after the i.v. injection of BPBBT NPs for 2 h. For BPBBT-HSA NPs, a great number of nanoparticle clusters were found heterogeneously distributed along the tumor vessels. Some nanoparticles extravasated from the vessels into the tumor interstitium (Figure 5A and Figure S4). By contrast, for BPBBT micelles and BPBBT-CL-HSA NPs, much fewer nanoparticles were observed to exist within or adjacent to the tumor endothelial cells. The quantitative analysis of the nanoparticle clusters along the tumor vessels confirmed that the endocytosed BPBBT-HSA NPs by the vessels was 5.21-fold and 6.01-fold of BPBBT micelles and BPBBT-CL-HSA NPs, respectively (Figure 5B). The extravasated BPBBT-HSA NPs was 18.83-fold and 16.95-fold of BPBBT micelles and BPBBT-CL-HSA NPs, respectively (Figure 5C). These results evidenced that the higher tumor accumulation of BPBBT-HSA NPs during the initial hours following i.v. administration observed in the biodistribution study was attributed to the active endothelial transcytosis. Cross-linking HSA resulted in the derecognition of the HSA nanoparticles by the endothelial cells with limited active transcytosis. Although PEGylation increased blood circulation and blood concentration of BPBBT micelles, PEG abated the interaction between the micelles and the endothelial cells, thus reducing the active transportation.

### 3.5. Extravasation of BPBBT NPs via Intravital Microscopic Imaging

#### 3.5.1. Endothelial Transcytosis of BPBBT-HSA NPs

**Figure 6:**
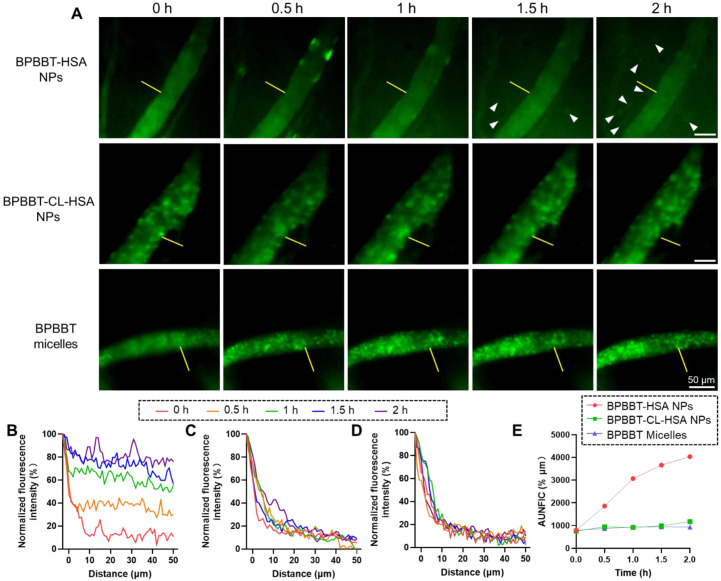
Intravital fluorescence imaging of the extravasation of different types of BPBBT NPs in the orthotopic CT26-Luc tumor of mice. (**A**) The distribution of BPBBT-HSA NPs (Video S1), BPBBT-CL-HSA NPs (Video S2) and BPBBT micelles (Video S3) in the CT26-luc tumor at different times after the injection, respectively. Green, the fluorescence signal of BPBBT. Arrows, the fluorescence signal of focal spots in the tumor interstitium. (**B**–**D**) Plots of the fluorescence intensity of BPBBT-HSA NPs (**B**), BPBBT-CL-HSA NPs (**C**) or BPBBT micelles (**D**) at different time points as a function of the distance from the blood vessel in a representative region marked by the yellow line in (**A**). (**E**) The area under the normalized fluorescence intensity-distance curve (AUNFIC) of BPBBT-HSA NPs, BPBBT-CL-HSA NPs and BPBBT micelles at different times post-injection, respectively.

We utilized the home-built intravital NIR-II fluorescence microscopic imaging system to visualize the dynamic process of extravasation of the nanoparticles into the tumor interstitium. At 0.5 h post administration of BPBBT-HSA NPs, the fluorescence of BPBBT in the tumor parenchyma started to appear and its intensity increased over time (Figure 6A,B, Video S1). It is noticed that the fluorescence of focal spots was observed in the tumor interstitium at 1.5 h post-injection (Figure 6A, arrows). The fluorescence spots became more intense at 2 h post-injection, suggesting that the albumin-bound nanoparticles were specifically taken up by the tumor cells following extravasation [41,42].

By contrast, in the mice injected with BPBBT-CL-HSA NPs (Figure 6A,C, Video S2) or BPBBT micelles (Figure 6A,D, Video S3), no obvious extravasation of the nanoparticles was observed during the initial 2 h following the injection. These comparative results supported that the denaturation of HSA or the PEGylation of the nanoparticles decreased the potential for endothelial cell ingestion as well as the efficiency of transcytosis. In order to compare the degree of extravasation of nanoparticles from the blood vessels to tumor parenchyma, we performed the integration of the normalized fluorescence intensity curves at each time point (Figure 6B–D), to calculate the values of AUNFIC. The results demonstrated that BPBBT NPs continuously improved the extravasation of nanoparticles in 2 h (Figure 6D). On the contrary, the values of AUNFIC in the BPBBT-CL-HSA NPs or BPBBT micelles group did not change significantly during the entirely observed time.

#### 3.5.2. Tumor Cell Uptake of BPBBT-HSA NPs

**Figure 7:**
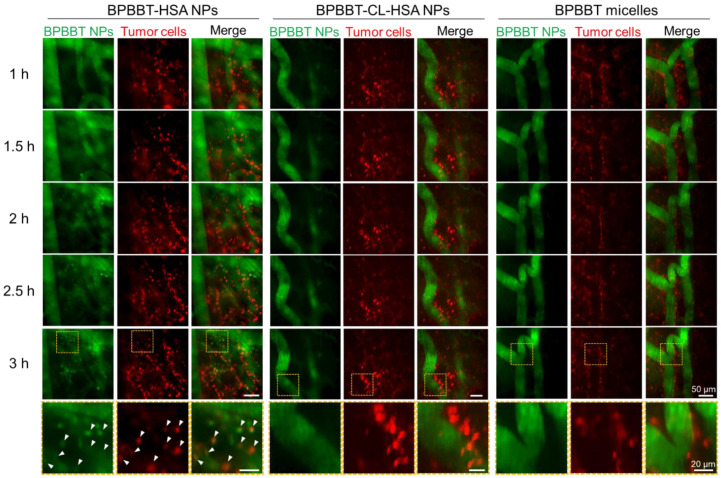
Intravital imaging of different types of BPBBT NPs in the orthotopic CT26-Luc tumor of mice after the i.v. injection (20 mg/kg of BPBBT). The tumor cells were labeled with DiD before the inoculation. Green, BPBBT NPs; red, the DiD-labeled tumor cells. The imaging started at 1 h and ended at 3 h post-injection of the nanoparticles. The box area of images at 3 h post-injection were enlarged and presented in the bottom row. Arrows, colocalization.

To investigate the process of BPBBT NPs endocytosed by the tumor cells, we labeled the tumor cells with DiD before the inoculation. The intravital NIR-II/Red dual channel fluorescence microscopic imaging was started at 1 h post-injection with different nanoparticles. In the group of BPBBT-HSA NPs, the focal spots of the fluorescence of BPBBT were observed in the tumor interstitium at 1.5 h post-injection. The number of the fluorescence spots increased over time and co-localized with the tumor cells (Figure 7, arrows, Video S4). However, BPBBT-CL-HSA NPs or BPBBT micelles showed little colocalization with the tumor cells (Videos S5 and S6). These results evidenced the tumor-targeted delivery effect of the albumin-bound nanoparticles.

## 4. Discussion

Intravital microscopic imaging has recently been extensively used to study the cell–cell interactions [43], the dynamic variation of the tumor microenvironment [44] and the biological process of nanoparticles, especially for their entry into solid tumors [17,19,42,45]. Compared to other traditional research techniques like immunohistochemistry (IHC) or TEM, intravital microscopic imaging allows tracking the nanoparticle delivery in real-time, enabling to describe the whole process of nanoparticles’ entry from the blood vessels into tumor interstitium and their uptake by tumor cells. In this study, we utilized the intravital NIR-II fluorescence microscopic imaging system to study the dynamic pathway for the nanoparticles’ entry into tumor. With regard to traditional intravital techniques, such as one-photon confocal or two-photon microscope, the limited imaging depth (<360 µm for conventional one-photon confocal imaging in the visible or NIR-I window) [32,46,47] and long acquisition time (1.25 to 5 s per frame) [19] restrain the in vivo application. Owing to the advantages of low spontaneous background fluorescence and tissue scattering interference, NIR-II fluorescence microscopic imaging could realize high imaging resolution with deep penetration (>1.3 mm) [32,48]. Furthermore, the epifluorescence imaging mode we chose had a short acquisition time (0.2 s per frame) without additional scanning time. The advantages of spatial and temporal resolution enable the NIR-II fluorescence microscopic imaging to be a suitable technique for real-time tracking the fate of nanoparticles in tumor.

Based on the EPR theory, nanoparticles extravasate through the inter-endothelial gaps into the tumor vasculature. The longer circulation time of nanoparticles contributes to the enhanced tumor accumulation. Herein, BPBBT-HSA NPs and BPBBT micelles with similar characteristics including particle size, PDI, drug loading, encapsulation efficiency and zeta potential were prepared for the comparison. Our results showed the PEGylation of BPBBT significantly improved the plasma elimination half-life and plasma concentration of BPBBT micelles. However, its contribution to the tumor-targeting efficiency was limited. Comparatively, the blood circulation time of BPBBT-HSA NPs was decreased to just about 1/3 of that of BPBBT micelles, while the AUC_tumor0→48h_ of BPBBT-HSA NPs was increased by 7.2-fold. The intravital NIR-II fluorescence microscopic imaging revealed that BPBBT-HSA NPs but not BPBBT micelles were transported by vascular endothelial cells with high efficiency, and captured by the tumor cells within 3 h. In addition, the tumor accumulation of BPBBT-HSA NPs was blocked by the cross-linking of the nanoparticles, evidenced by that the AUC_tumor0→48h_ of BPBBT-CL-HSA NPs was decreased by 78%. It should be mentioned that after the cross-linking by glutaraldehyde, the zeta potential of BPBBT-CL-HSA NPs was decreased from −16.97 mV to −25.40 mV, which was confirmed by bovine serum albumin nanoparticles [49]. Previous study compared the tumor distribution of the different rhodamine B-labeled carboxymethyl chitosan grafted nanoparticles (RhB-CMCNPs) of 150 nm or 500 nm in particles sizes with zeta potentials of −15, −25 or −40 mV [50]. The results showed that compared with the 150 nm-sized RhB-CMCNPs with −15 mV, the tumor distribution of RhB-CMCNPs of the same size with −25 mV was slightly decreased [50]. Instead, the particle size played a pivotal role in the tumor accumulation of RhB-CMCNPs. Thus, in our study the 8.43 mV of reduction may not be the major factor contributing to such huge decease of the tumor accumulation of BPBBT-CL-HSA NPs. Taken together, by using the extensively used nanoparticles like HSA NPs and micelles other than inorganic nanoparticles, our comparative study demonstrated that the tumor-targeting mechanism relies little on the EPR effect. The nanoparticles, especially the albumin-bound nanoparticles, are more likely to be transported in an active process by endothelium. In order to enhance the tumor-targeting rate, the designing of nanoparticles may be focused on how to improve the transcytosis efficiency. This assertion needs to be carefully verified by other types of nanoparticles in the future study.

## 5. Conclusions

In this work, we used BPBBT, the NIR-II fluorescence probe to investigate the tumor-targeting efficiency of three different types of nanoparticles, i.e., BPBBT-HSA NPs, BPBBT-CL-HSA NPs and BPBBT micelles. Given the similar particle sizes of ~110 nm, they showed different tumor-targeting efficiency. Compared with BPBBT-HSA NPs, although BPBBT micelles exhibited a longer systemic circulation time (*t*_1/2β_ = 10.00 v.s. 3.58 h), the micelles contributed much less to tumor accumulation, leading to their AUC_tumor0→48h_ only 14% of that of BPBBT-HSA NPs. The intravital microscopic imaging revealed the extravasation of BPBBT-HSA NPs from the tumor vasculature into tumor parenchyma with high efficiency within 3 h post-injection. However, the AUC_tumor0→48h_ of BPBBT-CL-HSA NPs was decreased to 22% of that BPBBT-HSA NPs. These results demonstrated that endothelial transcytosis was the dominant pathway for albumin-bound nanoparticles’ entry into tumor parenchyma. Our findings increased the understanding of active transport via endothelial transcytosis versus passive process through the EPR effect. In future design, it is important to consider more on nanomaterial engineering or tumor endothelium manipulation to enhance trans-endothelial transport so as to improve tumor-targeting efficiency. Our research provides a new paradigm for the design of nanomedicine for tumor-targeted delivery.

## Figures and Tables

**Table 1:** Characterization of different types of BPBBT NPs ^a^.

Types	Size (nm)	PDI	Zeta Potential (mV)	EE%	DL%
BPBBT-HSA NPs	109.67 ± 5.41	0.15 ± 0.01	−16.97 ± 0.75	79.67 ± 3.21	21.67 ± 3.06
BPBBT-CL-HSA NPs	108.07 ± 4.29	0.20 ± 0.01	−25.40 ± 1.91 *	73.33 ± 5.50	19.33 ± 3.06
BPBBT micelles	112.03 ± 8.88	0.13 ± 0.01	−17.47 ± 0.65	83.67 ± 5.51	23.67 ± 4.51

^a^ Data are presented as mean ± SD (*n* = 3). * *p* < 0.05 compared with the BPBBT-CL-HSA NPs group. Statistical significance was calculated by two-way ANOVA with Tukey’s post hoc test.

**Table 2:** Pharmacokinetic parameters of BPBBT after i.v. injection of BPBBT-HSA NPs, BPBBT-CL-HSA NPs or BPBBT micelles (20 mg/kg of BPBBT) into the tumor-bearing mice ^a^.

Parameters	BPBBT-HSA NPs	BPBBT-CL-HSA NPs	BPBBT Micelles
*t*_1/2α_ (h)	0.45 ± 0.33	0.30 ± 0.08	0.45 ± 0.25
*t*_1/2β_ (h)	3.58 ± 0.38	3.56 ± 0.34	10.00 ± 0.98 *
*Cl* (mL/h)	0.37 ± 0.02	0.51 ± 0.09	0.087 ± 0.01 **
AUC_0-t_ (μg/mL h)	805.06 ± 44.32	601.50 ± 110.82	3434.82 ± 260.73 **
*V*_ss_ (mL)	1.61 ± 0.15	2.35 ± 0.13 ^#^	1.21 ± 0.09

^a^ Data are presented as mean ± SD (*n* = 3). * *p* < 0.05, ** *p* < 0.01 compared with the BPBBT-HSA NPs group and BPBBT micelles group, ^#^ *p* < 0.05 compared with BPBBT-HSA NPs or BPBBT-CL-HSA NPs group. Statistical significance was calculated by two-way ANOVA with Tukey’s post hoc test.

## Data Availability

The data presented in this study are available on request from the corresponding author.

## Supplementary Materials

The following supporting information can be downloaded at: https://www.mdpi.com/article/10.3390/pharmaceutics15020519/s1, Figure S1: Chemical structure of BPBBT. Figure S2: UV-vis-NIR absorption spectra and fluorescence emission spectra (excited at 830 nm) of different types of BPBBT NPs in aqueous solution or BPBBT in THF solution (10 µM of BPBBT). Figure S3: Cell viability of NIH 3T3 cells after incubation with different BPBBT NPs at various concentrations for 24 h. Figure S4: (A) and (B) Representative immunofluorescence micrographs of tumor sections from additional two mice in Figure 5A, respectively; Video S1: Intravital NIR-II fluorescence micro-imaging of BPBBT-HSA NPs in CT26-Luc orthotopic tumor of mice following the i.v. injection; Video S2: Intravital NIR-II fluorescence micro-imaging of BPBBT-CL-HSA NPs in CT26-Luc orthotopic tumor of mice following the i.v. injection; Video S3: Intravital NIR-II fluorescence micro-imaging of BPBBT micelles in CT26-Luc orthotopic tumor of mice following the i.v. injection; Video S4: Intravital NIR-II/Red dual-channel fluorescence micro-imaging of BPBBT-HSA NPs in the DiD-labeled CT26-Luc orthotopic tumor of mice following the i.v. injection; Video S5: Intravital NIR-II/Red dual-channel fluorescence micro-imaging of BPBBT-CL-HSA NPs in the DiD-labeled CT26-Luc orthotopic tumor of mice following the i.v. injection; Video S6: Intravital NIR-II/Red dual-channel fluorescence micro-imaging of BPBBT micelles in the DiD-labeled CT26-Luc orthotopic tumor of mice following the i.v. injection.
