# Supplementary material for: Intravital Microscopy Reveals Endothelial Transcytosis Contributing to Significant Tumor Accumulation of Albumin Nanoparticles
Source: Pharmaceutics. 2023 Feb 3;15(2):519. doi: 10.3390/pharmaceutics15020519 (PMC9960641; doi:10.3390/pharmaceutics15020519)
Supplement: Supplementary file 1 [file pharmaceutics-15-00519-s001.zip › pharmaceutics-2164348-supplementary.pdf]

pharmaceutics

MDPI

Supplementary materials

Intravital Microscopy Reveals Endothelial Transcytosis Contributing to Significant Tumor Accumulation of Albumin Nanoparticles

Guoguang Wei, Sihang Zhang, Sheng Yu, Wei Lu*

Supplementary figures

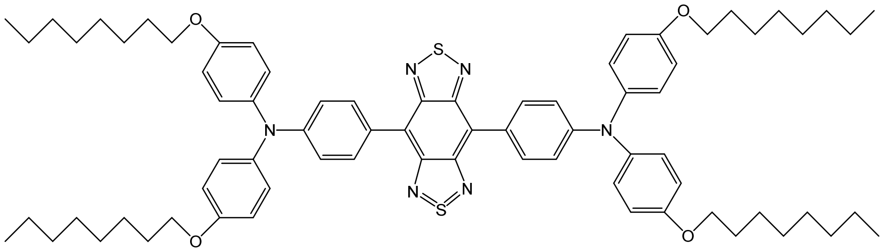

Figure S1. Chemical structure of BPBBT.

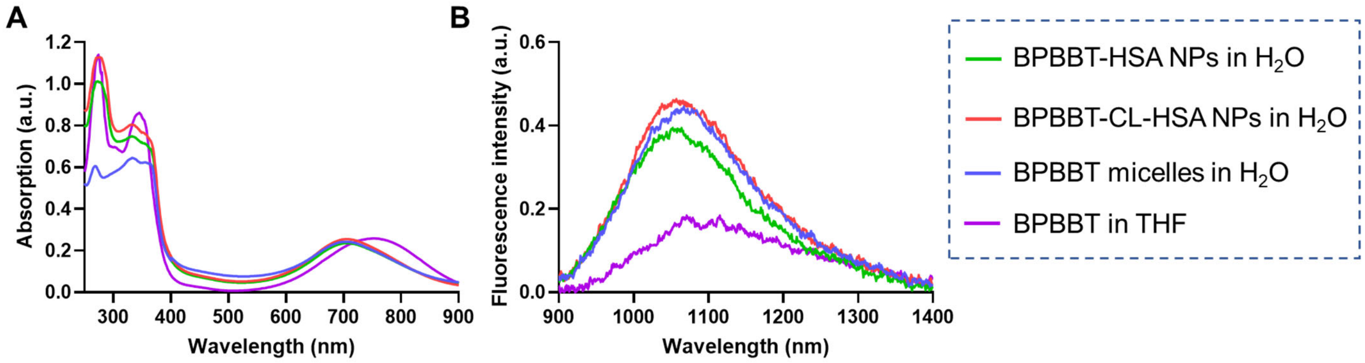

Figure S2. (A) UV-vis-NIR absorption spectra of different types of BPBBT NPs in aqueous solution or BPBBT in THF (10 μM of BPBBT). (B) Fluorescence emission spectra of different types of BPBBT NPs in aqueous solution or BPBBT in THF (10 μM of BPBBT) excited at 830 nm.

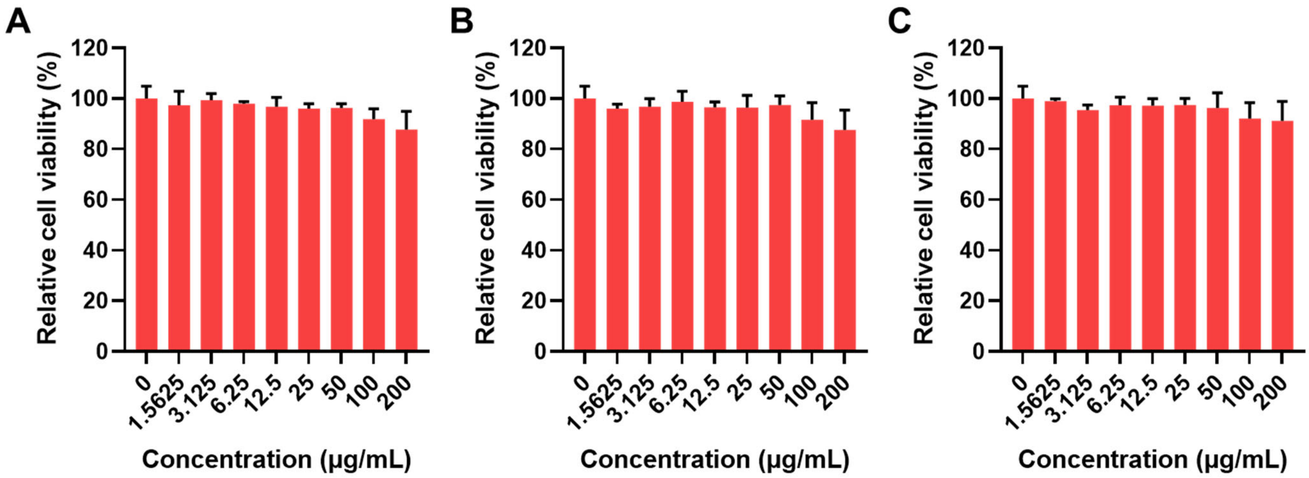

Figure S3. Cell viability of NIH 3T3 cells after incubation with (A) BPBBT-HSA NPs, (B) BPBBT-CL-HSA NPs or (C) BPBBT micelles at various concentrations for 24 h by MTT assay (*n* = 4). Data are presented as Mean ± S.D.

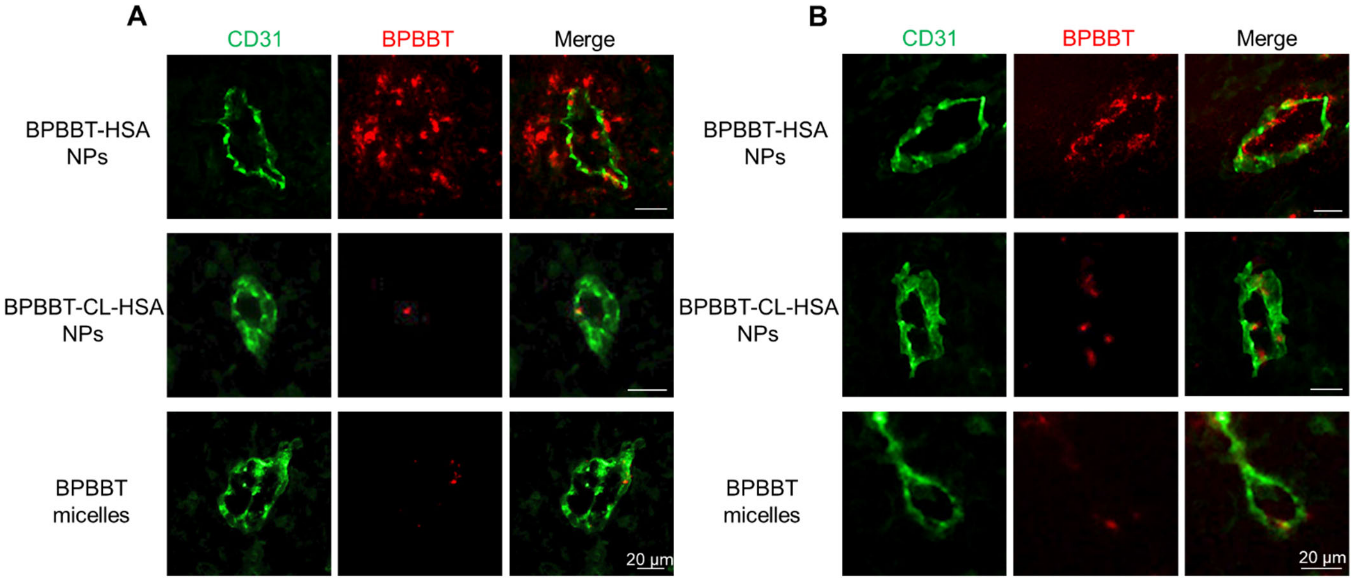

Figure S4. (A, B) Representative immunofluorescence micrographs of tumor sections from additional two mice in Figure 5A, respectively.

Supplementary video captions

Video S1. Intravital NIR-II fluorescence micro-imaging of BPBBT-HSA NPs in CT26-Luc orthotopic tumor of mice following the i.v. injection. The imaging started immediately after the injection.

Video S2. Intravital NIR-II fluorescence micro-imaging of BPBBT-CL-HSA NPs in CT26-Luc orthotopic tumor of mice following the i.v. injection. The imaging started immediately after the injection.

Video S3. Intravital NIR-II fluorescence micro-imaging of BPBBT micelles in CT26-Luc orthotopic tumor of mice following the i.v. injection. The imaging started immediately after the injection.

Video S4. Intravital NIR-II/Red dual-channel fluorescence micro-imaging of BPBBT-HSA NPs in the DiD-labeled CT26-Luc orthotopic tumor of mice following the i.v. injection. Green, BPBBT; Red, the DiD-labeled CT26-Luc tumor cells. The imaging started at 1 h post-injection of the nanoparticles.

Video S5. Intravital NIR-II/Red dual-channel fluorescence micro-imaging of BPBBT-CL-HSA NPs in the DiD-labeled CT26-Luc orthotopic tumor of mice following the i.v. injection. Green, BPBBT; Red, the DiD-labeled CT26-Luc tumor cells. The imaging started at 1 h post-injection of the nanoparticles.

Video S6. Intravital NIR-II/Red dual-channel fluorescence micro-imaging of BPBBT micelles in the DiD-labeled CT26-Luc orthotopic tumor of mice following the i.v. injection. Green, BPBBT; Red, the DiD-labeled CT26-Luc tumor cells. The imaging started at 1 h post-injection of the nanoparticles.
